# Supplementary material for: miR-252 targeting temperature receptor CcTRPM to mediate the transition from summer-form to winter-form of Cacopsylla chinensis
Source: eLife. 2023 Nov 15;12:RP88744. doi: 10.7554/eLife.88744 (PMC10651175; doi:10.7554/eLife.88744)
Supplement: Supplementary file 1. — (a) The primers used in current study. (b) Effect of agomir-252 and antagomir-252 treatment on the transition percent of SF 1st instar nymphs under 10 °C. [file elife-88744-supp1.docx]

**Supplementary file 1**

**miR-252 targeting temperature receptor *CcTRPM* to mediate the transition from summer-form to winter-form of *Cacopsylla chinensis***

Songdou Zhang, Jianying Li, Dongyue Zhang, Zhixian Zhang, Shili Meng, Zhen Li, Xiaoxia Liu^*^

Department of Entomology and MOA Key Lab of Pest Monitoring and Green Management, College of Plant Protection, China Agricultural University, 100193 Beijing, China

^*^ Corresponding author. E-mail address: liuxiaoxia611@cau.edu.cn (Xiaoxia Liu).

**Supplementary file 1a. The primers used in current study.**

| **Gene name**  **(Abbreviation)** | | **Accession number** | **Sequences (5’-3’)** | **Production length (bp)** | **Purpose** | **Amplification efficiency** |
| --- | --- | --- | --- | --- | --- | --- |
| *CcTRPM* | | OQ658558 | Full-F1: GGATAGAGGCAACTTTTCAA | 1307 | RT-PCR for sequence validation | — |
|  |  |  | Full-R1: ATCTATTCTGTCGTGCTCCA |  |  |  |
|  |  |  | Full-F2: TAGACGAAGCCATGATGC | 1162 | RT-PCR for sequence validation | — |
|  |  |  | Full-R2: ACAACTGTTTCACGGAGTGT |  |  |  |
|  |  |  | Full-F3: TAGCCAGACATTCTCAACAA | 1436 | RT-PCR for sequence validation | — |
|  |  |  | Full-R3: GAAGCATTACTATGGGTTGC |  |  |  |
|  |  |  | Full-F4: AGCAGACCCTTTCCACTC | 1334 | RT-PCR for sequence validation | — |
|  |  |  | Full-R4: TAACTGTTAACACATTGTTTCTGC |  |  |  |
|  |  |  | qF: ACAATGCATTCTTCTTGACC | 141 | qRT-PCR | 102.57 |
|  |  |  | qR: GACTTTGTACACTGGGGGTA |  |  |  |
|  |  |  | RNAi-F1: GATCACTAATACGACTCACTATAGGGAGA  GGTACCTTCGTATCCTCAAC | 472 | dsRNA synthesis | — |
|  |  |  | RNAi-R1: GATCACTAATACGACTCACTATAGGGAGA  TGGCATTGATCTCCATGAAA |  |  |  |
|  | |  | Probe: Cy3-CATAGATCGCGTAGGAGGTC | 20 | FISH | — |
|  |  |  | 3’UTR-Full-F1: CTAGTTGTTTAAACGAGCTC  CATTATGTAACCACAGCGTAA | 419 | Amplification of 3’UTR full sequence | — |
|  |  |  | 3’UTR-Full-R1: TGCATGCCTGCAGGTCGACTCTAGA  TTCCTAGAAACTCAAAAATCG |  |  |  |
|  |  |  | 3’UTR-Mut-F1: CTAGTTGTTTAAACGAGCTC  CATTATGTAACCACAGCGTAA | 137 | Amplification of 3’UTR mutant sequence | — |
|  |  |  | 3’UTR-Mut-R1: TGCATGCCTGCAGGTCGACTCTAGA  AAGTACTATCCACGCTCGAC |  |  |  |
|  |  |  | Orf-F: GGACGAGCTGTACAAGTAGGGATCC  ATGGAGAGGTCCCAGAGGTC | 4618 | Amplification of orf sequence | — |
|  |  |  | Orf-R: GTTTAAACGGGCCCTCTAGACTCGAGC  TTAACACATTGTTTCTGCGC |  |  |  |
| miR-252 | - | | qF: GCTAAGTACTGGTGCCGCAGGA | — | qRT-PCR | — |
| U6 | - | | qF: AGGATGACACGCAAAATCGT | — | qRT-PCR | — |
| *CcTre1* | OQ734934 | | qF: GGAACTCCCTCCTCTATGTT | 147 | qRT-PCR | 97.73 |
|  |  |  | qR: CCAATAGTCAGCCAATCTGT |  |  |  |
|  |  |  | RNAi-F1: GATCACTAATACGACTCACTATAGGGAGA  AGATGTCAAAACCAATCCAG | 455 | dsRNA synthesis | — |
|  |  |  | RNAi-R1: GATCACTAATACGACTCACTATAGGGAGA  GGCCAAGTTGTAGTATTTGC |  |  |  |
| *CcTre2* | OQ734935 | | qF: CCAATCCTTCTCTACACCTG | 123 | qRT-PCR | 93.68 |
|  |  |  | qR: CTGTCATTGTTGTACGGTTG |  |  |  |
| *CcTre-like* | OQ734936 | | qF: GACCGAACCTATGATCTCAA | 135 | qRT-PCR | 106.13 |
|  |  |  | qR: AGATGTACCAAGCTGCTGAG |  |  |  |
| *CcCHS1* | | OQ658570 | F: AGAAGAGAAGAAACAGCAGG | 233 | qRT-PCR | 101.47 |
|  |  |  | R: TAGTGTCCAATCGTTTCTCC |  |  |  |
|  |  |  | RNAi-F1: GATCACTAATACGACTCACTATAGGGAGA  TTGGCTACCACTGAGAAACT | 367 | dsRNA synthesis | — |
|  |  |  | RNAi-R1: GATCACTAATACGACTCACTATAGGGAGA  ATGCGTTTCGTACTCGTAAT |  |  |  |
| *CcCHS2* | | OQ658569 | F: TATTAGGCTGATGTTGCTGA | 124 | qRT-PCR | 95.49 |
|  |  |  | R: GAGGCAACACAAGAGGTAAG |  |  |  |
| *CcHK1* | | OQ658562 | F: GAGTCTGCCATCTACTGTCC | 130 | qRT-PCR | 96.21 |
|  |  |  | R: CGGGGGTGATATTTGTATAG |  |  |  |
| *CcHK2* | | OQ658560 | F: AAATAGTCCGACTGGCATTA | 137 | qRT-PCR | 98.05 |
|  |  |  | R: TGTACTCTTGGGCTCACTTT |  |  |  |
| *CcHK3* | | OQ658561 | F: AGCATTTGAGAGAGAACGTG | 120 | qRT-PCR | 103.51 |
|  |  |  | R: CCGCTGATATTGTCTTCTTC |  |  |  |
| *CcG6PI* | | OQ658563 | F: CCTGCTATCTCTCACAATCC | 127 | qRT-PCR | 107.23 |
|  |  |  | R: CTTTCAGTTCAGCTTCTGCT |  |  |  |
| *CcGFAT* | | OQ658564 | F: ACTGAAACATGGACCTCTTG | 123 | qRT-PCR | 97.06 |
|  |  |  | R: CTTCCATCTCTGGCAATAAC |  |  |  |
| *CcGNA* | | OQ658565 | F: GGCGAGTATTTTGTGAGTGT | 145 | qRT-PCR | 108.46 |
|  |  |  | R: CTCGGTACGTATCATCGACT |  |  |  |
| *CcAGM* | | OQ658566 | F: TTTATTTTGAGGCAAATGGT | 138 | qRT-PCR | 102.84 |
|  |  |  | R: AAGAGTCTCCGACAGTTTCA |  |  |  |
| *CcUAP1* | | OQ658567 | F: TGGGGAACTTGTGTATAACC | 124 | qRT-PCR | 98.03 |
|  |  |  | R: TGGAATCTTCTTGAGTGCTT |  |  |  |
| *CcUAP2* | | OQ658568 | F: ACATTGACTCCAAAGGATTG | 134 | qRT-PCR | 89.74 |
|  |  |  | R: GGCAAAATCATGTTCTCTGT |  |  |  |
| *CcEF-1* | | OQ658572 | qF: TGTGTCGAGTCATTCTCTGA | 124 | Reference gene for qRT-PCR | 98.63 |
|  |  |  | qR: TTACTTTACCGGAGGACAGA |  |  |  |
| *Ccβ-Actin* | | OQ658571 | qF: CAGCACCATGAAGATCAAG | 129 | Reference gene for qRT-PCR | 102.87 |
|  |  |  | qR: GACTCGTCATACTCCTGCTT |  |  |  |
| *EGFP* | | ACY56286 | F: GATCACTAATACGACTCACTATAGGGAGA  CTCCAGCAGGACCATGTGATC | 596 | dsRNA synthesis | — |
|  |  |  | R: GATCACTAATACGACTCACTATAGGGAGA  CCTGAAGTTCATCTGCACCAC |  |  |  |

Note: The black boxes indicated the T7 promoter sequences in the primers of dsRNA synthesis. The underlines showed the homologous arm sequences for the seamless cloning in homologous recombination.

**Supplementary file 1b. Effect of agomir-252 and antagomir-252 treatment on the transition percent of SF 1st instar nymphs under 10°C.**

| **Treatments** | **agomir-NC** | **agomir-252** | **dsEGFP** | **dsCcTRPM** | **dsCcTRPM+antagomir-252** |
| --- | --- | --- | --- | --- | --- |
| Average percent | 84.11 ± 8.37 | 38.89 ± 9.03 | 87.00 ± 5.79 | 31.56 ± 6.58 | 76.00 ± 7.78 |
